# Supplementary material for: End Sequence Analysis Toolkit (ESAT) expands the extractable information from single-cell RNA-seq data
Source: Genome Res. 2016 Oct;26(10):1397–410. doi: 10.1101/gr.207902.116 (PMC5052061; doi:10.1101/gr.207902.116)
Supplement: Supplemental Material [file supp_gr.207902.116_Supp_Materials.tar.gz › ESAT-esat_v0.1/doc/html/classumms_1_1core_1_1model_1_1_alignment_model_1_1_filtered_iterator-members.html]

ESAT: Member List


ESAT


- Main Page
- Related Pages
- Packages
- Classes
- Files

- Class List
- Class Index
- Class Hierarchy
- Class Members

All Classes Namespaces Files Functions Variables Enumerator Pages

- umms
- core
- model
- AlignmentModel
- FilteredIterator

umms.core.model.AlignmentModel.FilteredIterator Member List

This is the complete list of members for umms.core.model.AlignmentModel.FilteredIterator, including all inherited members.

|  |  |  |
| --- | --- | --- |
| close() | umms.core.model.AlignmentModel.FilteredIterator |  |
| FilteredIterator(CloseableIterator< AlignmentCount > overlappers, Annotation region, CoordinateSpace cs, boolean fullyContained) | umms.core.model.AlignmentModel.FilteredIterator |  |
| FilteredIterator(CloseableIterator< AlignmentCount > reads) | umms.core.model.AlignmentModel.FilteredIterator |  |
| hasNext() | umms.core.model.AlignmentModel.FilteredIterator |  |
| next() | umms.core.model.AlignmentModel.FilteredIterator |  |
| remove() | umms.core.model.AlignmentModel.FilteredIterator |  |


---

Generated on Fri Aug 15 2014 15:11:11 for ESAT by  

 1.8.7
